# Blueberry Pruning Wastes: From an Undervalued Agricultural Residue to a Safe and Valuable Source of Antioxidant Compounds for the Food Industry

**DOI:** 10.3390/foods13020317

**Published:** 2024-01-19

**Authors:** Olena Dorosh, Virgínia Cruz Fernandes, Cristina Delerue-Matos, Manuela M. Moreira

**Affiliations:** REQUIMTE/LAQV, Instituto Superior de Engenharia do Porto, Instituto Politécnico do Porto, Rua Dr. António Bernardino de Almeida, 431, 4249-015 Porto, Portugal; olena.dorosh@graq.isep.ipp.pt (O.D.); cmm@isep.ipp.pt (C.D.-M.); manuela.moreira@graq.isep.ipp.pt (M.M.M.)

**Keywords:** blueberry canes, circular economy, microwave-assisted extraction, subcritical water extraction, environmental contaminants, safety screening

## Abstract

Blueberry fruits have been widely explored for their rich composition of bioactive compounds with recognized health benefits. In contrast, blueberry pruning waste (BPW), generated during the pruning stages of blueberries, has been typically overlooked, even though it can represent a potential source of natural antioxidants. This study aims to characterize the value-added compounds extracted from BPW using green techniques, namely microwave-assisted and subcritical water extraction. The total phenolic content ranged from 157 ± 5 to 335 ± 12 mg GAE/g dw, while the radical scavenging activity determined by a DPPH assay varied from 223 ± 21 to 453 ± 21 mg Trolox equivalents/g dw. Additionally, to ensure the safe application of BPW and its extracts, a screening of pesticides and several environmental contaminants was conducted. Chlorpyrifos-methyl was quantified at a concentration of 4.27 µg/kg in a Bluecrop variety collected in 2019; however, none of the studied compounds were found in the extracts. Despite the presence of a pesticide, this level was below the maximum residue limits for blueberry crops. The results of this study demonstrated the potential of this agro-industrial residue as a natural source of bioactive compounds with high antioxidant activity for food industry applications.

## 1. Introduction

Blueberries (*Vaccinium sect. Cyanococcus*) are extremely rich in phenolic compounds, mainly anthocyanins, flavonols and chlorogenic acid, that possess a wide range of biological activities [1,2]. In fact, due to the presence of these secondary plant metabolites, some studies indicate that blueberries’ consumption may help to suppress inflammation and consequently decrease the risk of developing cancer, type 2 diabetes, arthritis and cardiovascular diseases [3,4]. These polyphenols’ health benefits are mainly derived from their antioxidant activity, which can help to neutralize free radicals in the body, in addition to their capacity to chelate transition metals and modify the redox potential of the cellular environment [5,6].

Currently, the major producers of blueberries are the United States of America and Canada [1]. These berries are highly appreciated worldwide as fresh fruits, as well as in juices, jams and yogurts, and incorporated as dried fruits in products like cereals, cookies and bars [1,2]. However, from the production of these fruit crops and their derivatives, a large quantity of residues is generated, including blueberry pomace, leaves and prunings. Over the past two decades, there has been a growing interest in the valorization of these agricultural and food processing wastes to obtain bioactive compounds [5]. Regarding the wastes generated from the use of blueberries, pomace and leaves have been the most studied ones [2,5,7,8]; however, as was observed for other fruits, the prunings could also represent an interesting source of phenolic compounds [9,10,11].

There are many techniques available for the extraction of phenolic compounds from agro-industrial foods and their by-products, mainly divided between conventional and environmentally friendly ones. Conventional extraction (CE) techniques (e.g., infusion, maceration, Soxhlet extraction, etc.) are the most practical and accessible in terms of laboratory equipment; nevertheless, they tend to be more time-consuming, use large amounts of organic solvents and produce diluted extracts [12,13]. This can represent a concern from both an environmental and economic perspective. Consequently, new techniques have been emerging, such as microwave-assisted extraction (MAE) and subcritical water extraction (SWE) [14,15]. These techniques allow the extraction time, energy consumption and amount of organic solvent used to be reduced. Further, all the previously mentioned extraction techniques can be up-scaled, representing an additional advantage in terms of industrial processes. In the case of the CE technique, it is considerably easier and cheaper to perform a scale-up of the extraction process. For MAE and SWE, it is possible to buy or custom order larger equipment from factories specialized in their production; however, their availability is still very scarce [15,16,17]. Despite the considerable literature available regarding the extraction of phenolic compounds from natural products, due to the large variability in matrixes, there is no standardized method [8]. In fact, many parameters play a role in the phenolic profile of extracts, such as the type of matrix, plant species, agricultural practices and storage conditions, as well as the extraction technique and the extraction parameters applied [1,7,8,18]. Some woody agro-industrial residues have already been investigated for the recovery of bioactive compounds, including those from berry plants [6,9,11]. Moreira et al. [11] were able to quantify up to 2936 mg of phloridzin/100 g dw by high-performance liquid chromatography (HPLC) coupled with a photodiode array detector (PAD) analysis of apple tree bark residue extracts obtained through CE and MAE. The extraction and isolation of this phenolic compound are highly interesting because it is associated with antidiabetic and antioxidant activities, the prevention of bone loss, memory enhancement and the inhibition of cancer cells [19,20]. Vine cane extracts obtained through SWE proved to be extremely rich in phenolic compounds with antioxidant activity [9]. Tian et al. [6] investigated the phenolic profile, antioxidant capacity and antibacterial activity of extracts obtained from Finnish berry plant leaves, prunings, pomace and the berry fruits themselves [6]. In this study, the values obtained for the DPPH free radical scavenging activity (DPPH-RSA) and oxygen radical absorbance capacity (ORAC) assays of the saskatoon (*Amelanchier alnifolia*) leaf branch extract were more promising than for the extract obtained from the respective berries. The saskatoon branch was also proven to inhibit the growth of *Escherichia coli* by 68%, *Bacillus aereus* by 84% and *Listeria monocytogenes* and *Staphylococus aureus* both by 100% when 20 µL was used in 300 µL of culture medium. The HPLC, coupled with a diode array detector (DAD) analysis of saskatoon berry and pruning extracts, presented differences in the classes of phenolic compounds identified for each matrix. The berry extract was mainly composed of phenolic acid derivatives, corresponding to 27.3 ± 0.5 mg/100 mL of the extract. However, the pruning extract was mainly composed by flavan-3-ols and proanthocyanidins, corresponding to 16.6 ± 0.9 and 21.1 ± 0.6 mg/100 mL of the extract. These two classes of phenolic compounds were probably the main contributors for the extracts’ antimicrobial activity [6]. While the extraction of bioactive compounds from agricultural by-products presents an environmental and economic opportunity, a critical challenge arises from the existing gap in the Maximum Residue Limits (MRLs) for the contaminants in these residues. Pesticides and heavy metals are commonly present on agricultural soils, crops and their by-products, either by direct application or absorption. However, while for crops there is strict regulation for the MRLs, there is still a need to establish them for their residues in order to systematically assess and manage potential risks, safeguarding both environmental health and consumer safety [21,22].

This study compared the blueberry pruning phenolic profiles and antioxidant activities of extracts obtained through CE, MAE and SWE from two different blueberry varieties, namely Duke and Bluecrop, collected in the 2019 and 2020 pruning seasons. Further, to evaluate the safety of these BPWs and their respective extracts, a screening of pesticides and environmental contaminants was also performed. This type of research has a big window of opportunity right now because consumers are becoming more demanding regarding the sustainability of food production and nutritional quality, increasing the search for more natural and safe sources of ingredients [1,23,24]. Therefore, these agro-industrial by-products could constitute a natural source of bioactive compounds to be incorporated in nutritional supplements, nutraceuticals, functional products, bioplastics, pharmaceuticals and cosmetic products.

This study was the first to comprehensively detail the phenolic composition and antioxidant activity of blueberry cane extracts. Furthermore, a notable gap was identified in the existing literature, with no prior studies investigating screening for pesticides and other environmental contaminants in blueberry residues. The results obtained revealed an intriguing residue rich in bioactive compounds that had not been previously explored.

## 2. Materials and Methods

### 2.1. Chemicals and Regents

The absolute ethanol (≥99%) used in the extractions was obtained from Fisher Chemical (Loughborough, UK). 2,2-Diphenyl-1-picrylhydrazyl radical (DPPH^•^), (±)-6-hydroxyl-2,5,7,8-tetramethylchromane-2-carboxylic acid (Trolox^®,^ Madrid, Spain), Folin–Ciocalteu’s (FC) phenol reagent and sodium carbonate (≥99%), used in the spectrophotometric assays, were purchased from Sigma-Aldrich (Madrid, Spain). 2,4,6-Tris(2-pyridyl)-s-triazine (TPTZ, 99%), (−)-epicatechin (≥90%), gallic acid monohydrate (GA, ≥98%) and hydrochloric acid (≥37%) were obtained from Fluka (Munich, Germany). Aluminum chloride (99.52%) and sodium acetate 3-hydrate (99%) were acquired from Panreac (Barcelona, Spain). Iron(III)chloride-6-hydrate (≥99%) and L(+)- ascorbic acid (AA, ≥99.7%) were from Riedel-de Haën (Seelze, German), glacial acetic acid (≥99.5%) was from Carlo Erba (Peypin, France), sodium nitrite (98%) was from M&B Chemicals (City Road, London, UK) and sodium hydroxide (>99%) was from Labkem (Zelienople, PA, USA). For the HPLC analysis, the methanol (≥99.8%) and formic acid (≥98%) gradient grades were from VWR (Lisboa, Portugal), respectively. Individual phenolic compound standards were purchased from Sigma-Aldrich (Madrid, Spain), and their purity was at least above 95%. Standards of brominated flame retardants (BFRs) with ≥98% purity were acquired from Isostandards Material, S.L. (Madrid, Spain) at 50 mg/L inisooctane and corresponded to 2,4,4′-tribromodiphenyl ether (BDE28); 2,2′,4,4′-tetrabromodiphenyl ether (BDE47); 2,2′,4,4′,5-pentabromodiphenyl ether (BDE99); 2,2′,4,4′,6-pentabromodiphenyl ether (BDE100); 2,2′,4,4′,5,5′-hexabromodiphenyl ether (BDE153); 2,2′,4,4′,5,6′-hexabromobiphenyl ether (BDE154); and 2,2′,4,4′,5,5′-hexabromodiphenyl ether (BDE183). Standards of organophosphorous pesticides (OPPs) (chlorfenvinphos, chlorpyrifos, chlorpyrifos-methyl, dimethoate, parathion-methyl and malathion) and of organochloride pesticides (OCPs), hexachlorobenzene (HCB); α-, β- and ζ- hexachlorocyclohexane (HCH); [1,1,1-trichloro- 2-(2-chlorophenyl)-2-(4-chlorophenyl)ethane] (o,p′-DDT); 2,2-bis(4- chlorophenyl)-1,1-dichloroethylene (p,p′-DDE); 1-chloro-4-[2,2- dichloro-1-(4-chlorophenyl)ethyl]benzene (p,p′-DDD); aldrin; dieldrin; α-endosulfan; methoxychlor; and lindane) with ≥99% purity were obtained from Sigma-Aldrich (St. Louis, MO, USA). The polychlorinated byphenyl (PCB) standards, 2,4,4′-trichlorobiphenyl (PCB28), 2,2′,4,5,5′-pentachlorobiphenyl (PCB101), 2,3′,4,4′,5-pentachlorobiphenyl (PCB118), 2,2′,4,4′,5,5′-hexachlorobiphenyl (PCB153) and 2,2′,3,4,4′,5,5′-heptachlorobiphenyl (PCB180), were purchased from Riedel-de Haën (Seelze, Germany) at 10 ng/μL in isooctane. The internal standards (ISs) 4,4′-dichlorobenzophone and triphenyl phosphate were from Sigma-Aldrich (St. Louis, MO, USA) and were used for the analysis of halogenated organic compounds and organophosphorus pesticides, respectively. Chromatographic-grade n-hexane and acetonitrile were acquired from UniSolv Merck (Darmstadt, Germany) and Carlo Erba (Val de Reuli, France), respectively. The commercial QuEChERS powders, the d-SPE and the SampliQ Carbon SPE Bulk Sorbent, were purchased from Agilent Technologies (Santa Clara, CA, USA). The Strata C 18-E SPE cartridges were obtained from Phenomenex (Madrid, Spain).

### 2.2. Sample Collection and Preparation

The BPW from the two different varieties, namely Duke and Bluecrop, were kindly provided by Mirtidão, Lda company, and sampled in Viseu (Portugal) in 2019 and 2020 by randomized selection. After arriving at the laboratory, the samples were milled (ZM200, Retsch, Porto, Portugal) and sieved (AS200 Basic, Tetsch, Porto, Portugal) to a size comprised between 710 µm and 1 mm and stored in sealed plastic bags until use. The moisture content was determined using a moisture analyzer (Kern MLS 50-3IR160, KERN and SOHN, Balingen, Germany). All the samples analyzed contain similar percentages of moisture, varying from 7.91 ± 0.36 to 9.67 ± 0.36% for the Duke variety in 2019 and 2020, respectively.

### 2.3. Extraction of Bioactive Compounds

#### 2.3.1. Conventional Extraction

For the CE, 0.5 g of milled BPW and 20 mL of a 50:50 *v*/*v* ethanol/water mixture were mixed in Erlenmeyer flasks, and the extraction was performed for 2 h at 55 °C in a water bath shaker (model BSC127E, C from OVAN) at 100 rpm, as previously described by Moreira et al. [11].

#### 2.3.2. Microwave-Assisted Extraction

MAE was performed with 0.1 g of milled BPW and 20 mL of a 60:40 *v*/*v* ethanol/water mixture for 20 min at 100 °C in the MARS-X 1500 W (Microwave-Accelerated Reaction System for Extraction and Digestion, CEM, Mathews, North Carolina, USA). The conditions applied were previously optimized by Moreira et al. [11] for vineyard prunings.

#### 2.3.3. Subcritical Water Extraction

SWE was conducted using a Parr Series 4560 Mini Reactor connected to a Parr 4848 Reactor Controller (Parr Instrument Company, Moline, IL, USA). The extraction was performed using 20 g of a sample and 200 mL of water at 280 °C for 32 min once the desired temperature was reached, with a constant stirring rate of 250 rpm. These conditions were previously optimized for vineyard pruning residues [25], changing the temperature range from 150 to 280 °C and the extraction time from 20 to 50 min, as defined by the Response Surface Model design.

All the extraction mixtures were centrifuged at 15,763× *g* (Heraeus Megafuge 16 Centrifuge Series, Thermo Scientific, Waltham, MA, USA) for 15 min at 4 °C. Afterwards, the extracts were stored at −80 °C and lyophilized (Edwards lyophilizer, Edwards Vacuum, Sussex, UK) for 48 h. The dried samples were stored in a dark and dry place until further use.

### 2.4. Determination of Total Phenolic and Flavonoid Contents

The total phenolic content (TPC) was determined using the Folin–Ciocalteau method, as previously described by Dorosh et al. [9]. GA was used as a standard (linearity range: 10–150 mg/L, R^2^ > 0.9994), and the results were expressed as mg of GA equivalents (GAE) per g of dry weight (dw) of extract (mg GAE/g dw). The total flavonoid content (TFC) was measured according to the aluminum chloride procedure also described by Dorosh et al. [9]. In this assay, epicatechin was used for calibration (linearity range: 5–150 mg/L, R^2^ > 0.9992), and the results were expressed as mg of epicatechin equivalents (EE) per g of dw (mg EE/g dw). Each extract was analyzed in triplicate.

### 2.5. Quantitative and Qualitative Polyphenol Characterization

The BPW extracts’ phenolic profiles were analyzed by high-performance liquid chromatography (HPLC) with photodiode array (PDA) detection. The analysis was conducted using an HPLC system (Shimadzu Corporation, Kyoto, Japan) with a 250 mm × 4.6 mm Gemini C_18_ column from Phenomenex (Madrid, Spain) and a guard column with the same characteristics that was kept at 25 °C, according to the method described by Pinto et al. [26]. The mobile phase was composed of methanol (A) and water (B), both acidified with 0.1% formic acid, and the elution gradient was carried out at a flow rate of 1.0 mL/min. A PDA at 280, 320 and 360 nm was used to detect and quantify the individual phenolic compounds. The extracts were prepared in methanol: water 20:80 *v*/*v* and filtered through a 0.22 μm-pore-sized nylon filter before injection. Standards were prepared in a 50:50 *v*/*v* methanol/water mixture in a linearity range of 1 to 200 mg/L. Each extract was analyzed three times, and the results were expressed as mg of compound/g of dw.

### 2.6. DPPH-RSA, FRAP and ABTS Assays

The DPPH-RSA, FRAP and ABTS assays were performed in order to evaluate the BPW extracts’ antioxidant activities. The methodology followed for the DPPH-RSA and FRAP assays was described in detail by Dorosh et al. [9]. Trolox was used for the DPPH-RSA calibration curve (linearity range: 10–150 mg/L, R^2^ > 0.9975), and the results were expressed as mg Trolox equivalents per g of dw (mg TE/g dw). For the FRAP assay, calibration was performed with AA (linearity range: 10–100 mg/L, R^2^ > 0.9989), and the results were expressed in mg of AA equivalents per g of dw (mg AAE/g dw). The ABTS protocol was performed as previously described by Pinto et al. [27]. The calibration curve (linearity range: 5–50 mg/L, R^2^ > 0.9994) was obtained using AA, and the results were expressed as mg AAE/g dw. Each extract was analyzed in triplicate.

### 2.7. Screening of Pesticides and Other Environmental Contaminants in BPWs and Their Extracts

The presence of environmental contaminants and pesticides was evaluated for the BPWs and their respective extracts. The sample preparation and result analysis for the BPW were performed according to the procedure optimized by Olena et al. [21]. For the experiment, 2 g of BPW was subjected to an original QuEChERS extraction, followed by a dispersive solid-phase extraction (d-SPE), prior to the gas chromatography (GC) analysis.

Regarding the BPW extracts, the analysis was performed based on the protocol described by Fernandes et al. [28], with slight modifications. For the solid-phase extraction, the solvents (*n*-hexane, methanol and ultra-pure water) were conditioned twice through the Strata C18-E cartridges, and then the extract, with a concentration of 1 mg/Ml, was loaded into the cartridges. Ultra-pure water was used to wash off any remaining impurities, and afterward, the samples were subjected to a vacuum for 10 min. Once dried, the extract was eluted with *n*-hexane, which was then evaporated under a nitrogen atmosphere (Reacti-therm III #TS-18823, Thermo Scientific). The resulting extract was redissolved in *n*-hexane and ready to be analyzed in the GC.

#### 2.7.1. OPP Compounds’ Gas Chromatography Analysis

A mixture containing 6 OPP standards (dimethoate, chlorpyrifos-methyl, parathion-methyl, malathion, chlorpyrifos and chlorfenvinphos) at a concentration of 50 µg/L was injected and analyzed. A GC with a flame photometric detector (GC-FPD) (GC-2010, Shimadzu, Quioto, Japan) using a phosphorus filter and a 30.0 m TRX-5 column with a 0.25 mm inner diameter and 0.25 µm film thickness from *Teknokroma* was used for the analysis of these environmental contaminants. Helium at 1 mL/min with a linear velocity of 25.4 cm/s was used as the carrier gas. The column oven’s starting temperature corresponded to 100 °C, which was kept for 1 min before increasing it to 150 °C at a rate of 20 °C/min, where it was held for 1 min again. Following this, the temperature was increased to 180 °C at 2 °C/min and kept for 2 min, and lastly increased at 20 °C/min to 270 °C, where it was kept for 1 min. The detector was at 250 °C in splitless mode, and the analytes were detected at 290 °C. The GCsolution Version 2.41.00 software used was Shimadzu’s GC Solution. Triphenyl phosphate (TPP) was used as the IS for the OPP analysis.

#### 2.7.2. OCP and PYR Gas Chromatography Analysis

In the GC with an electron capture detector (GC-ECD) (GC-2010, Shimadzu, Quioto, Japan), a mixture of 14 OCP (α-HCH, HCB, β-HCH, lindane, ζ-HCH, aldrin, endosulfan I, o,p’-DDT, dieldrin, endrin, endosulfan II, p,p’-DDD, p,p’-DDE and methoxychlor), 7 PYR (bifenthrin, cyhalothrin, permethrin I, permethrin II, cyfluthrin, cypermethrin, fenvalerate I, fenvalerate II and deltamethrin), 5 PCB (PCB28, PCB101, PCB118, PCB153 and PCB180) and 7 BDE (BDE28, BDE47, BDE100, BDE99, BDE154, BDE153 and BDE183) standards with a concentration of 50 µg/L was injected. The column used was a 30.0 m Zebron XLB column with a 0.25 mm inner diameter and a 0.25 μm film thickness from *Phenomenex*. The carrier gas was helium injected at 1.09 mL/min with a linear velocity of 26.6 cm/s, and nitrogen injected at 30 mL/min was used as the make-up gas. Injections were performed using a splitless mode with an injector temperature of 250 °C, and the detector temperature was set at 300 °C. At the beginning of the injection, the column oven was maintained at 40 °C for 1 min, increasing to 250 °C at a rate of 30 °C/min, where it was kept for 11 min. Afterwards, the temperature was increased at a rate of 10 °C/min to 290 °C, where it was kept for 9 min. The GC software used was the same as the one used for the OPP data analysis. Dichlorobenzophenone (DCBP) was used as the IS for the organochloride pesticide (OCP) and pyrethroid (PYR) analyses.

### 2.8. Statistical Analysis

The statistical analysis was performed using GraphPad Prism 8.0 software. To determine significant differences among the multiple groups, Tukey’s multiple comparisons test was employed. This post hoc test allows for a comparison of all the possible pairs of means, effectively identifying any statistically significant differences between the groups following an analysis of variance (ANOVA).

## 3. Results and Discussion

### 3.1. Total Phenolic and Flavonoid Contents

The comprehensive assessment of the TPC and TFC within the Duke and Bluecrop BPW varieties collected in 2019 and 2020, encompassing extracts obtained through CE, MAE and SWE, is summarized in Table 1.

Regarding the TPC, statistically significant differences (*p* < 0.05) were evident among the applied extraction techniques as well as between the years of sampling. On the other hand, the differences between the blueberry varieties were not so evident. The highest values corresponded to the extracts from the Duke and Bluecrop varieties from 2019 obtained by the CE technique (335 ± 12 and 333 ± 23 mg GAE/g dw, respectively). On the other hand, the lowest TPC value was obtained for the Bluecrop variety from 2020 subjected to MAE, corresponding to 157 ± 5 GAE/g dw. For the TFC, statistically significant differences (*p* < 0.05) were also noticed between the extracts obtained through CE and those prepared through MAE and SWE. These findings align with those obtained for the TPC, where CE facilitated the attainment of the highest results. However, unlike the TPC results, marginal disparities between MAE and SWE were observed for the TFC. This suggests that flavonoids may not be the specific subclass of phenolic compounds contributing significantly to the variations observed in the TPC. The highest value registered for the TFC was 217 ± 17 mg EE/g dw for the Bluecrop variety collected in 2019 and subjected to CE, and the lowest was 105 ± 5 for the Bluecrop variety collected in 2020 and subjected to MAE. These findings diverged from our prior research, which had previously identified CE as the least effective extraction technique for the recovery of polyphenols from vineyards and apple tree prunings [11,26]. A potential hypothesis to explain this phenomenon is that the phenolic compounds mostly present in the BPW can be more thermolabile. Hence, the application of higher temperatures, such as the ones used in MAE and SWE, could have led to the degradation of a greater number of compounds during the extraction process [29]. In fact, a study performed by Khanal et al. [30] demonstrated that heating significantly decreased the procyanidin and anthocyanin concentrations in blueberry pomace at temperatures above 60 °C. In addition, the observed differences between the BPW sampled in different years may be related to biotic and abiotic stress factors, often prompting an increased production of phenolic compounds as a natural defense mechanism against external aggressions [31].

No studies reporting the extraction of phenolic compounds from blueberry canes were found in the literature. However, other by-products derived from blueberry processing have already been investigated for the obtention of bioactive compounds [1,7,32]. Additionally, the prunings of other fruits and berries were also explored [9,11,26]. Lončarić et al. [32] employed high-voltage electrical discharges (HVEDs), pulsed electric fields (PEFs) and ultrasound-assisted extraction (UAE) on blueberry pomace. Extractions were performed with hydroalcoholic solvents (50% ethanol and 1% HCl) in a 1:50 solid-to-liquid ratio (g/mL). The highest TPC reported by the authors was 10.52 mg GAE/g dw for the PEF technique, which was considerably lower than the values reported for the blueberry canes. Bamba et al. [1] optimized phenolic compound extraction from blueberry pomace using UAE, achieving the highest results with a solid-to-liquid ratio of 1:20 (g/mL), 50% ethanol, at 40 °C for 60 min. These optimized conditions allowed them to extract 35.95 ± 0.12 mg GAE/g dw for the TPC and up to approximately 20 mg mg EE/g dw for the TFC. The collective findings from both studies [1,32] suggest that blueberry canes may serve as a superior source of phenolic compounds, or alternative extraction methodologies may be more efficient than those employed by the respective authors. Deng et al. [7] explored the utilization of blueberry leaves for the extraction of phenolic compounds. The authors blended leaf powders with 95% aqueous ethanol at a solid-to-liquid ratio of 15:100 (g/mL), maintaining a temperature of 50 °C for 30 min. Under these conditions, the TPC obtained was approximately 80 mg GAE/g dw, significantly lower than the phenolic content reported for the blueberry canes (Table 1). However, by employing solely methanol at a solid-to-liquid ratio of 1:10 (g/mL) and a temperature of 25 °C for 30 min, the authors were able to extract 349 mg GAE/g dw. This result demonstrates that blueberry leaves indeed constitute a rich source of phenolic compounds, but the conditions applied using ethanol were suboptimal for their extraction. Notably, Bamba et al. [1] conducted a comprehensive series of extractions with ethanol concentrations ranging from 10% to 90% and concluded that 50% ethanol was the most favorable for extracting phenolic compounds from blueberry pomace, and possibly the same could be transferred for the leaves. Previous research from the group that performed CE and MAE on apple tree prunings and CE, MAE and SWE on vine prunings reported considerably lower results than the ones obtained for the blueberry canes [9,11,26]. The highest values registered for the apple tree prunings were 44.4 ± 1.1 mg GAE/g dw and 20.3 ± 1.2 mg EE/g dw for the TPC and TFC assays, respectively [11]. The highest TPC, 181 ± 12 mg GAE/g dw, reported by the group for the vine canes was obtained through SWE and was in fact higher than the one obtained for the blueberry canes subjected to SWE, but still lower than the extracts obtained through CE and MAE (Table 1). The highest TFC reported for the vine canes was 51 ± 6 mg EE/g dw, and it was also using SWE [9]. These results suggest that the pruning of berries represents a more attractive source of phenolic compounds than the pruning of fruit trees such as apples.

### 3.2. Antioxidant Activity

Regarding the antioxidant activity, the BPW extracts obtained through CE exhibited the highest DPPH-RSA and ABTS, aligning with their superior TPCs and TFCs. In contrast, the BPW subjected to SWE exhibited the lowest values for these assays. Notably, for the FRAP assay, the highest values were registered for the samples obtained through SWE. These variations may be attributed to the distinct mechanisms of action employed by the assays. The ABTS and DPPH-RSA assays assess a sample’s capacity to donate hydrogen atoms, while the FRAP assay evaluates its ability to reduce ferric (Fe^3+^) to ferrous (Fe^2+^) ions by donating electrons. Consequently, the bioactive compounds present in the samples resultant from CE can easily donate hydrogen atoms, while the ones resultant from SWE can easily donate electrons [33]. The highest DPPH-RSA activity was 453 ± 21 and 452 ± 36 mg TE/g dw, and the highest ABTS was 468 ± 17 and 457 ± 36 AAE/g dw, both for the Duke and Bluecrop varieties sampled in 2019 and subjected to CE. The highest value registered for the FRAP assay was 359 ± 21 mg AAE/g dw for the Duke variety sampled in 2020.

There are also no studies available in the literature that measured the antioxidant capacity of BPW extracts; however, these assays have already been performed on other blueberry residues and prunings from other fruits [1,9,11,26]. In a study conducted by Bamba et al. [1], blueberry pomace extracts obtained through UAE were able to obtain an antioxidant activity of 64.25 ± 0.39 mg TE/g dw in a DPPH-RSA assay. These results significantly lag behind those obtained from the blueberry canes for any of the extraction methods used (Table 1), which was also observed for the TPC and TFC. Once more, this suggests that either the raw material, the extraction technique used by Bamba et al. [1] or both do not allow values as high as the ones reported for the blueberry canes to be obtained.

In previous studies performed by the group using the same extraction techniques but different matrixes, Moreira et al. [11] reported the highest antioxidant capacity for apple tree prunings as 29.6 ± 1.3 TE/g dw and 39.8 ± 2.0 mg AAE/g dw for the DPPH-RSA and FRAP assays, respectively. In contrast to the BPW, the antioxidant activity of the apple pruning extracts obtained through MAE exceeded that obtained through CE. Regarding extractions performed using vine canes [9,26], the highest values were reported by Olena et al. [9]. The authors were able to obtain an antioxidant activity of 203 ± 22 mg TE/g dw and 202 ± 14 mg AAE/g dw for the DPPH-RSA and FRAP assays, respectively. The BPW presented higher antioxidant activities than any of the extracts obtained from the apple and vine prunings, with the exception of the antioxidant activity exhibited by the blueberry canes subjected to MAE for the FRAP assay.

The results obtained in the previous assays demonstrate that BPW is an extremely rich source of phenolic compounds with antioxidant properties and can represent an interesting raw material to be re-used by other industries. In general, the BPW extracts from the Bluecrop variety sampled in 2020 presented the lowest values, while the Bluecrop variety from 2019 presented the highest. Further, the most promising results were obtained for the CE, demonstrating the importance of testing diverse extraction techniques to exploit the most efficient one to recover bioactive compounds from different biomass materials.

### 3.3. Identification and Quantification of Polyphenols

**Figure 1:**
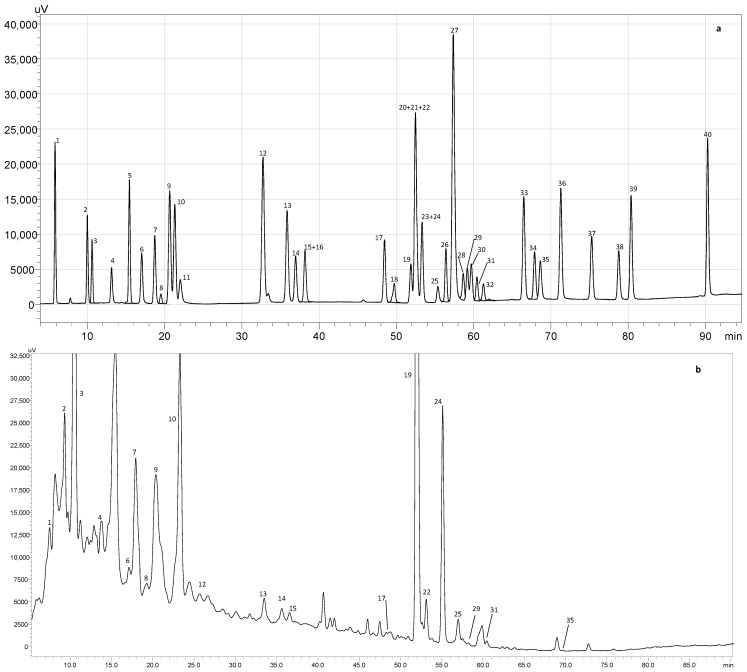
HPLC-PDA chromatogram monitored at 280 nm for (**a**) polyphenol standard mixture of 5 mg/L and (**b**) conventional extract from BPW from Duke variety from 2019; peak identification: (1) gallic acid, (2) protocatechuic acid, (3) neochlorogenic acid, (4) (+)-catechin, (5) caftaric acid, (6) caffeine, (7) chlorogenic acid, (8) 4-O-caffeyolquinic acid, (9) vanillic acid, (10) caffeic acid, (11) syringic acid, (12) (−)-epicatechin, (13) p-coumaric acid, (14) trans-ferulic acid, (15) sinapic acid, (16) trans-polydatin, (17) naringin, (18) 3,5-di-caffeoylquinic acid, (19) quercetin-3-O-galactoside, (20) resveratrol, (21) quercetin-3-O-glucopyranoside, (22) rutin, (23) phloridzin, (24) ellagic acid, (25) 3,4-di-O-caffeoylquinic acid; (26) myricetin, (27) cinnamic acid, (28) quercitrin, (29) kaempferol-3-O-glucoside, (30) isorhamnetin-3-O-glucoside, (31) kaempferol-3-O-rutinoside, (32) isorhamnetin-3-O-rutinoside, (33) naringenin, (34) trans-*ε* viniferin, (35) quercetin, (36) phloretin, (37) tiliroside, (38) kaempferol, (39) apigenin and (40) chrysin.

The BPW extracts obtained by CE, MAE and SWE were further subjected to an HPLC-PDA analysis to elucidate the key phenolic compounds responsible for conferring antioxidant properties upon the extracts. The identified and quantified compounds are presented in Table 2, and Figure 1 shows a representative HPLC-PDA chromatogram at 280 nm for one of the obtained extracts.

Notably, the highest quantities of phenolic compounds quantified corresponded to 232 ± 12 and 223 ± 11 mg/g dw for the Duke and Bluecrop varieties, respectively, both sampled in 2019 and subjected to CE. These results align with the TPCs and TFCs shown in Table 1, where the extracts of BPW sampled in 2019 and obtained using CE exhibited the highest values of phenolic compounds. Additionally, these extracts displayed remarkable antioxidant activity, as assessed by the DPPH-RSA and ABTS assays (Table 1).

Phenolic acids were the major class of compounds identified and quantified in the samples (Table 2), varying between 43% and 56% for the SWE Bluecrop 2020 and MAE Duke 2019 samples, respectively. The primary phenolic acids extracted using the CE and MAE techniques were neochlorogenic and protocatechuic acids, while those extracted using SWE were 4-*O*-caffeoylquinic, caftaric, chologenic, gallic and vanillic acids. Deng et al. [7] identified and quantified some of the phenolic compounds present in the extracts of blueberry leaves obtained through CE with 95% ethanol. The authors [7] identified and quantified the gallic, *p*-coumaric, ferulic, caffeic, vanillic and syringic acids (1.020 ± 0.000, 0.864 ± 0.010, 1.260 ± 0.348, 9.034 ± 0.000, 9.727 ± 1.648 and 4.609 ± 0.000 mg/g dw) in the blueberry leaf extracts obtained through CE, obtaining values very similar to the ones obtained for the BPW using CE (Table 2). However, the protocatechuic acid quantified (0.847 ± 0.000) was notably lower, thereby constituting one of the compounds that significantly contribute to the substantial disparity observed in the TPC values obtained between the studies. Lončarić et al. [32] identified and quantified chlorogenic and caffeic acids in the blueberry pomace extracts. The highest amount of chlorogenic acid was quantified for the samples obtained through HVED and corresponded to 0.569 ± 0.005 mg/g dw. The highest amount of caffeic acid was quantified for the samples obtained using PEF, corresponding to 0.0069 ± 0.0002 mg/g dw. Both phenolic acids were quantified in considerably higher quantities in the BPW, which is in accordance with the results obtained for the TPC, suggesting that the matrix or the extraction techniques used in the present study, or even both, are more advantageous for the extraction of phenolic compounds.

Flavanols, representing another significant class of phenolic compounds, were also found in substantial quantities within the BPW extracts obtained using conventional and subcritical water extractions (Table 2). An interesting difference between these two extraction techniques was that when using CE, similar quantities of catechin and epicatechin were extracted, whereas when using SWE, the catechin compound corresponded to the largest percentage of flavanols extracted. For the vine canes [9,26], catechin was also quantified in considerably higher amounts than epicatechin for the extracts obtained through SWE. In one case, it was almost 250-fold higher, suggesting that this extraction technique greatly favors the extraction of catechin. The highest amount of flavanols extracted from the vine canes using SWE (5.94 ± 0.29 mg/g dw) was below the amount of flavanols quantified for any of the blueberry cane extracts. For the BPW extracts obtained via SWE, flavanols accounted for a notable percentage, ranging from 36% to 40% of the total quantified phenolic compounds for the Duke variety collected in 2019 and Bluecrop variety collected in 2020, respectively. These proportions closely resembled the levels of phenolic acids quantified, suggesting that these two classes of phenolic compounds contributed in a comparable manner to the antioxidant activities of the extracts obtained through SWE. Lončarić et al. [32] were able to quantify 0.059 ± 0.02 and 0.075 ± 0.03 mg/g dw of catechin and epicatechin for the blueberry pomace extracts obtained through PEF. Once again, the results obtained in the present study were considerably higher (Table 2).

Within the flavonols class, the compound quercetin-3-*O*-galactoside should be highlighted, which was also quantified in substantial amounts for the BPW extracts prepared using the CE and MAE techniques, representing approximately 30% of the total phenolic compounds quantified for the BPW extract from the Duke variety sampled in 2019. This compound is associated with several potential health benefits, such as (a) the neutralization of free radicals in the body, reducing oxidative stress and inflammation; (b) anti-inflammation through the modulation of inflammatory pathways; (c) a reduction in blood pressure and cholesterol levels; (d) an enhancement of the immune system; (e) neuroprotective potential, reducing the risk of neurodegenerative diseases; and (f) allergy relief through the inhibition of histamine release [34].

Each blueberry sample (each variety and each year of collection) was submitted to three different extraction techniques, and all of the extracts presented a variation in the type and amount of phenolic compounds extracted (Table 2). As the samples were the same, these variations were caused by the extraction techniques. Indeed, the results obtained by Moreira et al. [11], who determined the phenolic composition of the extracts from apple tree wood residues prepared by different extraction techniques, agree with our findings. These authors reported differences in the phenolic profiles and amounts of each compound, which were dependent on the extraction technique applied. According to the authors, the use of different temperatures plays a pivotal role during the extraction process, as higher temperatures can lead to the degradation of some thermolabile compounds. Further, the use of different solvents, which possess varying affinities for specific compounds, can also result in the selective extraction of certain components over others, justifying the higher efficiency of some extraction techniques [34].

In a previous study conducted by Moreira et al. [26], the same extraction techniques used in this work were applied to vine canes, which also belong to the berry plant family. These authors reported that the primary compounds extracted from vine canes using the MAE and SWE techniques were gallic acid, followed by catechin, myricetin, quercetin, kaempferol and resveratrol. Even though vine canes are from different plant sources, some of the predominant compounds were the same as those reported in the present study. However, when comparing the results from the two studies, it can be concluded that BPW may represent a more promising agro-industrial residue to exploit, as the lowest total phenolic compound content quantified by HPLC-PDA was 87.0 mg/g dw versus 15.0 mg/g dw for the vine canes.

The present results underscore the remarkable abundance of phenolic compounds present in BPW and demonstrate that this under-valorized residue can have economical value for the obtention of natural bioactive compounds. In future work, it would be interesting to study the antibacterial activity of the blueberry cane extracts, since it was already reported in the literature that blueberry phenolics were efficient in decreasing bacterial cell auto-aggregation and motility and affecting cellular hydrophobicity [35]. Considering the abundance of phenolic compounds in the blueberry cane extracts analyzed, it is very probable that they also contain these antibacterial characteristics and therefore could be used as preservatives in food and cosmetic products.

### 3.4. Screening of Pesticides and Environmental Contaminants in Blueberry Canes and Extracts

Once the potential of the blueberry canes regarding their phenolic content and antioxidant capacity was determined and demonstrated, a safety assessment of the samples was performed. The presence of environmental contaminants, specifically persistent organic pollutants (POPs) like PCB and BDE, in both food and environmental samples is a pressing concern due to their long-term persistence, bioaccumulation and potential adverse effects on human health and ecosystems [21,36]. Therefore, the goal was to understand if any of these POPs were present in the canes and, most importantly, in the extracts.

From the chromatogram analysis of the samples, it was possible to quantify the presence of chlorpyrifos-methyl at a concentration of 4.27 µg/kg in the Bluecrop variety collected in 2019. For this sample, the limit of detection corresponded to 0.50 µg/kg and the limit of quantification to 1.68 µg/kg. Even though there are no regulations for the limit of pesticides and environmental pollutants in BPW, according to annexes II and III that were added to Regulation (EC) No. 396/2005 of the European Parliament and of the Council, the limit allowed in blueberry fruits is 0.01 mg/kg, which means that the amount quantified in the BPW is below the established limit [37]. Regarding the BPW extracts, no contaminants were found in any of the extracts, which indicates that the chlorpyrifos-methyl present in the Bluecrop variety collected in 2019 did not pass from the matrix to the solvent during the extraction process. In 2019, when the first sampling of the blueberry canes was conducted, the use of chlorpyrifos-methyl was still allowed in the European Union; however, its use was banned in 2020 due to its effects on the environment and human health. Some of these effects include: (a) toxicity, acute exposure can lead to nausea, dizziness, headaches and, in severe cases, respiratory paralysis or death; (b) neurotoxicity, due to overstimulation of the nervous system; (c) developmental and reproductive effects, exposure to chlorpyrifos-methyl during pregnancy may be associated with developmental and reproductive effects in both animals and humans; (d) endocrine disruption, this compound may interfere with the hormonal system in both humans and wildlife; and (e) environmental persistence, chlorpyrifos-methyl has the potential to persist in the environment, leading to concerns about its impact on non-target organisms, including wildlife such as birds, fish and insects. Runoff from treated fields can also contribute to water contamination [38]. Hence, even if in minimal quantities, the absence of contaminants on blueberry canes and, particularly, in the extracts is highly preferable. No existing studies provide a benchmark for pesticide determinations in blueberry residues or extracts, precluding direct comparisons. Nevertheless, Milinčić et al. [39] conducted a screening for fifteen pesticides in blueberries from Serbia, detecting nine pesticides in concentrations ranging from 5.15 µg/kg for thiametoxan to 187 µg/kg for azoxytrobin. Since none of these pesticides were investigated in our samples, subjecting Duke and Bluecrop BPW and extracts to this analysis would offer valuable insights. Notably, in the United States, blueberries were recently included in the “Dirty Dozen” list of the 2023 Shopper’s Guide to Pesticides in Produce because they contain 54 different types of pesticides, including phosmet and malathion [40]. This list was compiled by the Environmental Working Group—a nonprofit environmental health organization comprising scientists, policy experts, lawyers and data specialists—whose mission is to educate consumers and encourage industries to adopt higher standards. Several of the pesticides identified in these blueberry samples are no longer permitted in the European Union, reducing the risks associated with consuming blueberries from EU countries.

The results obtained for the BPW and their extracts suggest that they are likely safe for incorporation into products intended for human consumption. While no POPs were detected in the analyzed blueberry cane extracts, the continuous monitoring and assessment of these contaminants in food and environmental matrices remains crucial. Such ongoing scrutiny is essential for understanding their prevalence, assessing potential health risks and implementing measures to mitigate their impact on both environmental and human health [41]. In future work, in vitro and in vivo studies should be performed in order to guarantee the safety of these extracts and the recommended dosages for food applications.

## 4. Conclusions

The findings presented in this study underscore the substantial richness of phenolic compounds with antioxidant properties within BPW. Extracts derived from this agro-industrial residue hold significant potential for serving as natural antioxidants in a wide range of industries, offering a sustainable alternative to synthetic compounds in applications such as food production, cosmetics and nutraceuticals, as well as in the formulation of paints, lubricants and fuels. One noteworthy observation of this work was that CE allowed us to extract higher quantities of phenolic compounds than the other two techniques tested (MAE and SWE). This enhanced performance is likely attributed to the comparatively lower temperatures employed during CE, thereby preserving the integrity of some thermolabile phenolic compounds. For future work, it would be interesting to test lower extraction temperatures when using environmentally friendly extraction techniques, such as the ones tested in the present work (MAE and SWE). The TPCs and the phenolic profiles of the extracts were found to be influenced by several factors, including the BPW variety, the year of sampling and the extraction technique applied. Thus, it is imperative to consider these variables when designing an optimal extraction protocol. Additionally, safety assessments were conducted for both the BPW varieties and their respective extracts. While a trace amount of chlorpyrifos-methyl at a concentration of 4.27 µg/kg was quantified in one of the BPW samples, no contaminants were found in the extracts.

In light of the increasing demand for environmentally friendly ingredients, the application of these BPW extracts not only aligns with this growing trend but also offers an innovative and sustainable solution to various industrial challenges. This approach offers an eco-friendly approach to obtaining valuable compounds and aligns with the growing interest in environmentally responsible practices in the industry.

## Figures and Tables

**Table 1:** Total phenolic content (TPC; results expressed in mg gallic acid equivalents (GAE)/g dw), total flavonoid content (TFC; results expressed in mg epicatechin equivalents (EE)/g dw), 2,2-diphenyl-1-picrylhydrazyl radical scavenging activity (DPPH-RSA; results expressed in milligram Trolox equivalents (TE)/g dw) and ferric reduction antioxidant power (FRAP; results expressed in milligram ascorbic acid equivalents (AAE)/g dw) of blueberry pruning waste (BPW) extracts obtained through conventional (CE), microwave-assisted (MAE) and subcritical water (SWE) extractions of the Duke and Bluecrop varieties collected in 2019 and 2020. Results were expressed as mean ± standard deviation (*n* = 3).

BPW Variety and Year	Extraction Technique	TPC * (mg GAE/g dw)	TFC * (mg EE/g dw)	DPPH-RSA * (mg TE/g dw)	FRAP * (mg AAE/g dw)	ABTS * (mg AAE/g dw)
Duke 2019	CE	335 ± 12 a	205 ± 17 a,b	453 ± 21 a	280 ± 4 b	468 ± 17 a
	MAE	193 ± 16 d,e	129 ± 11 c,d	258 ± 3 c,d,e	175 ± 1 d	257 ± 19 d,e
	SWE	241 ± 11 c	123 ± 12 c,d	226 ± 13 d,e	345 ± 15 a	300 ± 11 c,d
Duke 2020	CE	286 ± 4 b	208 ± 10 a	354 ± 23 b	255 ± 5 c	412 ± 25 a,b
	MAE	187 ± 4 e	128 ± 2 c,d	269 ± 20 c,d	154 ± 5 d	234 ± 21 e
	SWE	243 ± 11 c	137 ± 5 c	223 ± 22 e	359 ± 21 a	281 ± 26 c,d
Bluecrop 2019	CE	333 ± 23 a	217 ± 17 a	452 ± 36 a	283 ± 17 b	457 ± 36 a
	MAE	199 ± 10 d,e	134 ± 14 c	287 ± 18 c	159 ± 11 d	256 ± 6 d,e
	SWE	213 ± 9 d	137 ± 12 c	230 ± 13 d,e	299 ± 7 b	281 ± 20 c,d
Bluecrop 2020	CE	277 ± 10 b	180 ± 15 b	385 ± 19 b	246 ± 7 c	427 ± 39 a,b
	MAE	157 ± 5 f	105 ± 8 d	225 ± 23 d,e	120 ± 2 e	224 ± 14 e
	SWE	248 ± 7 c	129 ± 10 c,d	254 ± 11 c,d,e	355 ± 7 a	312 ± 14 c

Different letters in the same column mean significant differences (*p* < 0.05) between samples. * Tukey’s multiple comparisons test.

**Table 2:** Content of the identified phenolic compounds in blueberry pruning waste extracts obtained through conventional (CE), microwave-assisted (MAE) and subcritical water (SWE) extractions of the Duke and Bluecrop varieties collected in 2019 and 2020. Results were expressed as mean ± standard deviations (mg of compound/g dw, *n* = 3).

Compound	Duke 2019			Duke 2020			Bluecrop 2019			Bluecrop 2020		
	CE	MAE	SWE	CE	MAE	SWE	CE	MAE	SWE	CE	MAE	SWE
***Phenolic acids***												
3,5-di-caffeoylquinic acid	<LOQ ^a^	<LOD ^b^	0.81 ± 0.04	<LOQ	<LOD	1.24 ± 0.06	<LOD	<LOD	0.68 ± 0.03	<LOD	<LOD	0.83 ± 0.04
4-O-caffeyolquinic acid	1.10 ± 0.06	0.57 ± 0.03	8.13 ± 0.41	0.60 ± 0.03	0.56 ± 0.03	15.11 ± 0.76	1.63 ± 0.08	2.47 ± 0.12	10.08 ± 0.50	<LOQ	<LOQ	9.47 ± 0.47
4,5-di-O-caffeoylquinic acid	1.18 ± 0.06	0.59 ± 0.03	0.87 ± 0.04	0.79 ± 0.04	<LOD	1.33 ± 0.07	0.75 ± 0.04	<LOD	0.84 ± 0.04	0.75 ± 0.04	<LOQ	0.65 ± 0.03
*p*-Coumaric acid	0.61 ± 0.03	0.84 ± 0.04	0.94 ± 0.05	<LOQ	<LOQ	1.55 ± 0.08	0.72 ± 0.04	<LOD	1.07 ± 0.05	<LOD	<LOQ	1.72 ± 0.09
Caffeic acid	8.79 ± 0.44	4.62 ± 0.23	1.83 ± 0.09	11.1 ± 0.5	6.25 ± 0.31	3.13 ± 0.16	14.5 ± 0.7	5.88 ± 0.29	2.14 ± 0.11	13.4 ± 0.7	6.03 ± 0.30	3.44 ± 0.17
Caftaric acid	<LOQ	<LOD	10.9 ± 0.5	<LOD	<LOD	19.7 ± 0.9	<LOQ	<LOQ	15.4 ± 0.8	<LOD	<LOD	16.1 ± 0.8
Chlorogenic acid	7.40 ± 0.37	4.37 ± 0.22	7.38 ± 0.37	4.40 ± 0.22	3.59 ± 0.18	14.3 ± 0.7	9.42 ± 0.47	<LOQ	8.68 ± 0.43	3.93 ± 0.20	3.58 ± 0.18	13.3 ± 0.7
Cinnamic acid	<LOD	ND ^c^	<LOD	<LOD	<LOD	<LOQ	ND	<LOD	<LOQ	<LOD	<LOD	0.79 ± 0.04
Ellagic acid	13.2 ± 0.7	7.58 ± 0.38	<LOQ	11.9 ± 0.6	6.49 ± 0.32	<LOD	12.9 ± 0.6	6.93 ± 0.35	3.46 ± 0.17	5.21 ± 0.26	5.20 ± 0.26	<LOD
Ferulic acid	1.41 ± 0.07	<LOQ	1.19 ± 0.06	<LOD	<LOD	2.00 ± 0.10	0.61 ± 0.03	<LOQ	1.41 ± 0.07	<LOD	<LOQ	2.22 ± 0.11
Gallic acid	1.48 ± 0.07	1.07 ± 0.05	8.32 ± 0.42	0.68 ± 0.03	0.61 ± 0.03	8.43 ± 0.42	2.18 ± 0.11	1.04 ± 0.05	5.03 ± 0.25	0.88 ± 0.04	0.72 ± 0.04	9.07 ± 0.45
Neochlorogenic acid	31.4 ± 1.6	14.4 ± 0.7	0.77 ± 0.04	26.6 ± 1.3	12.9 ± 0.6	1.77 ± 0.09	16.7 ± 0.8	13.9 ± 0.7	1.29 ± 0.06	19.8 ± 0.9	7.49 ± 0.37	1.13 ± 0.06
Protocatechuic acid	30.8 ± 1.5	17.9 ± 0.9	1.26 ± 0.06	31.0 ± 1.5	14.2 ± 0.7	<LOD	22.1 ± 1.1	18.5 ± 0.9	<LOD	20.8 ± 1.0	9.18 ± 0.46	<LOQ
Sinapic acid	4.32 ± 0.22	0.64 ± 0.03	4.11 ± 0.21	<LOD	<LOD	6.95 ± 0.35	<LOD	<LOQ	5.45 ± 0.27	<LOD	<LOD	7.73 ± 0.39
Syringic acid	ND	1.71 ± 0.09	1.64 ± 0.08	3.32 ± 0.17	1.51 ± 0.08	1.21 ± 0.06	4.07 ± 0.20	1.39 ± 0.07	0.77 ± 0.04	4.24 ± 0.21	1.34 ± 0.07	1.26 ± 0.06
Vanillic acid	8.64 ± 0.43	4.06 ± 0.20	10.4 ± 0.5	6.95 ± 0.35	4.31 ± 0.22	14.7 ± 0.7	10.9 ± 0.5	3.01 ± 0.15	9.22 ± 0.46	10.4 ± 0.5	4.74 ± 0.24	14.6 ± 0.7
**∑Phenolic acids**	110 ± 6	58.5 ± 2.9	58.6 ± 2.9	97.4 ± 4.9	50.4 ± 2.5	91.4 ± 4.6	96.6 ± 4.8	53.2 ± 2.7	65.5 ± 3.3	79.4 ± 4.0	38.3 ± 1.9	82.3 ± 5.1
***Flavanols***												
Catechin	21.5 ± 1.1	3.09 ± 0.15	34.8 ± 1.7	11.7 ± 0.6	7.92 ± 0.40	62.5 ± 3.1	25.2 ± 1.3	13.1 ± 0.7	45.1 ± 2.2	16.7 ± 0.8	7.88 ± 0.39	62.9 ± 3.1
Epicatechin	20.5 ± 1.0	6.28 ± 0.31	8.33 ± 0.42	8.01 ± 0.40	3.89 ± 0.19	15.0 ± 0.7	9.96 ± 0.50	1.57 ± 0.08	10.1 ± 0.5	10.82 ± 0.54	3.76 ± 0.19	16.5 ± 0.8
**∑Flavanols**	41.9 ± 2.1	9.37 ± 0.46	43.2 ± 2.2	19.7 ± 0.9	11.8 ± 0.6	77.5 ± 3.9	35.2 ± 1.8	14.7 ± 0.7	55.2 ± 2.7	27.49 ± 1.37	11.6 ± 0.6	79.4 ± 3.9
***Flavanones***												
Naringenin	ND	<LOD	<LOD	<LOD	<LOD	<LOQ	<LOD	<LOD	<LOQ	<LOD	ND	0.83 ± 0.04
Naringin	0.83 ± 0.04	<LOD	2.81 ± 0.14	<LOD	<LOD	5.29 ± 0.26	<LOQ	<LOD	4.06 ± 0.20	<LOQ	<LOD	7.02 ± 0.35
**∑Flavanones**	0.83 ± 0.04	0.0	2.81 ± 0.14	0.0	0.0	5.29 ± 0.26	0.0	0.0	4.06 ± 0.20	0.0	0.0	7.85 ± 0.39
***Flavonols***												
Isorhamnetin-3-O-rutinoside	<LOQ	<LOD	<LOD	1.71 ± 0.09	<LOQ	1.04 ± 0.05	0.70 ± 0.03	1.11 ± 0.06	<LOD	1.46 ± 0.07	0.78 ± 0.04	<LOD
Kaempferol-3-O-glucoside	1.86 ± 0.09	0.68 ± 0.03	<LOD	1.38 ± 0.07	0.60 ± 0.03	<LOQ	1.05 ± 0.05	0.71 ± 0.04	1.00 ± 0.05	1.11 ± 0.06	<LOQ	ND
Kaempferol-3-o-rutinoside	1.27 ± 0.06	0.76 ± 0.04	0.85 ± 0.04	0.90 ± 0.04	<LOQ	<LOD	1.64 ± 0.08	<LOQ	0.76 ± 0.04	0.68 ±0.03	<LOQ	2.31 ± 0.12
Myricetin	<LOQ	<LOD	3.43 ± 0.17	0.52 ± 0.03	0.35 ± 0.02	6.29 ± 0.31	<LOQ	<LOQ	5.25 ± 0.26	<LOQ	<LOQ	8.03 ± 0.40
Quercetin	0.62 ± 0.03	<LOQ	<LOQ	<LOQ	<LOQ	ND	0.95 ± 0.05	<LOQ	<LOQ	<LOQ	<LOQ	0.73 ± 0.04
Quercetin-3-O-galactoside	67.1 ± 3.4	36.9 ± 1.8	3.70 ± 0.18	59.5 ± 2.9	32.9 ± 1.7	6.27 ± 0.31	62.4 ± 3.1	34.5 ± 1.7	<LOQ	55.7 ± 2.8	26.1 ± 1.3	8.90 ± 0.44
Quercetin-3-O-glucopyranoside	<LOQ	<LOQ	<LOQ	14.2 ± 0.7	8.35 ± 0.42	0.51 ± 0.03	14.6 ± 0.7	8.50 ± 0.42	ND	13.3 ± 0.7	6.62 ± 0.33	0.74 ± 0.04
Rutin	2.35 ± 0.12	1.41 ± 0.07	0.53 ± 0.03	ND	ND	1.15 ± 0.06	ND	ND	<LOQ	ND	ND	0.80 ± 0.04
**∑Flavonols**	73.1 ± 3.7	39.8 ± 1.9	8.51 ± 0.42	78.2 ± 3.9	42.3 ± 2.1	15.3 ± 0.8	81.3 ± 4.1	44.7 ± 2.2	7.01 ± 0.35	72.3 ± 3.5	33.5 ± 1.7	21.5 ± 1.1
**∑Flavonoids**	115.83 ± 5.84	49.17 ± 2.36	54.52 ± 2.76	97.9 ± 4.8	54.1 ± 2.7	98.09 ± 4.96	116.5 ± 5.9	59.4 ± 2.9	66.27 ± 3.25	99.79 ± 4.87	47.1 ± 2.3	108.75 ± 5.39
***Stilbenes***												
Resveratrol	<LOQ	<LOQ	<LOQ	2.40 ± 0.12	1.41 ± 0.07	0.69 ± 0.03	2.49 ± 0.12	1.46 ± 0.07	<LOQ	2.24 ± 0.11	1.14 ± 0.06	<LOQ
*trans*-Polydatin	<LOD	<LOD	0.62 ± 0.03	<LOD	<LOD	1.10 ± 0.06	ND	<LOD	0.56 ± 0.03	<LOD	<LOQ	<LOD
**∑Stilbenes**	0.0	0.0	0.0	2.40 ± 0.12	1.41 ± 0.07	0.69 ± 0.03	2.49 ± 0.12	1.46 ± 0.07	0.0	2.24 ± 0.11	1.14 ± 0.06	0.0
***Others***												
Caffeine	5.82 ± 0.29	2.91 ± 0.15	2.27 ± 0.11	3.94 ± 0.20	2.68 ± 0.13	6.15 ± 0.31	7.38 ± 0.37	2.06 ± 0.10	4.13 ± 0.21	4.86 ± 0.24	2.45 ± 0.12	4.21 ± 0.21
Phloridzin	<LOD	<LOD	2.73 ± 0.14	<LOD	ND	2.85 ± 0.14	ND	<LOD	4.67 ± 0.23	ND	ND	4.12 ± 0.21
**∑Others**	0.0	0.0	3.35 ± 0.17	0.0	0.0	3.95 ± 0.20	0.0	0.0	5.23 ± 0.26	0.0	0.0	4.12 ± 0.21
**∑All phenolic compounds**	232	110	119	202	109	200	223	116	141	186	87.0	199

^a^ LOQ, limit of quantification; ^b^ LOD, limit of detection.; ^c^ ND, not detected.

## Data Availability

Data is contained within the article.
